# Transmission Heterogeneity and Control Strategies for Infectious Disease Emergence

**DOI:** 10.1371/journal.pone.0000747

**Published:** 2007-08-22

**Authors:** Luca Bolzoni, Leslie Real, Giulio De Leo

**Affiliations:** 1 Dipartimento di Scienze Ambientali, Università degli Studi di Parma, Parma, Italy; 2 Department of Biology, Emory University, Atlanta, Georgia, United States of America; University of Liverpool, United Kingdom

## Abstract

**Background:**

The control of emergence and spread of infectious diseases depends critically on the details of the genetic makeup of pathogens and hosts, their immunological, behavioral and ecological traits, and the pattern of temporal and spatial contacts among the age/stage-classes of susceptible and infectious host individuals.

**Methods and Findings:**

We show that failing to acknowledge the existence of heterogeneities in the transmission rate among age/stage-classes can make traditional eradication and control strategies ineffective, and in some cases, policies aimed at controlling pathogen emergence can even increase disease incidence in the host. When control strategies target for reduction in numbers those subsets of the population that effectively limit the production of new susceptible individuals, then control can produce a flush of new susceptibles entering the population. The availability of a new cohort of susceptibles may actually increase disease incidence. We illustrate these general points using Classical Swine Fever as a reference disease.

**Conclusion:**

Negative effects of culling are robust to alternative formulations of epidemiological processes and underline the importance of better assessing transmission structure in the design of wildlife disease control strategies.

## Introduction

Historically, models for infectious diseases considered populations of host and pathogen to be well-mixed with homogeneous disease transmission among susceptible and infected individuals. In recent years, however, we have recognized that transmission may not be constant but may vary with time, social structure, and/or age/stage-class. In human epidemiology it is well known that transmission may indeed change substantially among social groups and age classes, as observed, for instance, in the early phases of HIV epidemics [1], [2]. Recent research has also emphasized the importance of super-spreaders in the dynamics of SARS, sexually transmitted and childhood diseases [3], [4]. However, there is only anecdotal information about variation in transmission rate by age/stage structure in wild animals or the implication of such variation on disease dynamics in zoonotic reservoirs [5] and only limited information in the case of domestic animals (see e.g., [6]). This is unfortunate since the majority of emerging infectious diseases are zoonotic and it is well know that many species exhibit a high degree of heterogeneity in their spatial, social and age/stage structure. It is highly likely that age/stage-dependent behavioral differences in reservoir contact rates may significantly affect disease transmission between and within different age/stage classes and, as a consequence, the effectiveness of disease control policies.

While the science of infectious diseases has made tremendous progress in the last several decades thanks, in part, to advances in molecular biology, immunology, medicine and mathematical modeling, the eradication of pathogens and parasites in wildlife relies very often only on two simple strategies, namely vaccination and culling, i.e., the removal of animals to push host population density below the threshold for disease invasion [7]. Quarantine and isolation through the construction of sanitary containments are rare options in the control of wildlife diseases and are applied primarily to domestic animals and farms such in the case of the foot-and-mouth epidemics in UK [8] and of avian flu epidemics in Asia [9] often at the cost of huge economic losses.

Oral vaccination, on the contrary, is a quite widespread technique for disease eradication and control: it has been applied in the USA to control the westward expansion of rabies virus in raccoon hosts and has effectively eliminated rabies in coyotes in Texas [10], [11]. In Germany, oral vaccination is used to control classical swine fever virus exposure in wild boars [12]. The drawbacks of oral vaccination mainly consist in the difficulty of producing, at a reasonable price, a sufficient amount of a vaccine able to persist long enough in baits so as to be picked up by a suitable fraction of susceptible animals. Moreover, vaccination might be less effective than expected, as older animals–who may have antibodies resulting from prior infection–are often more aggressive in ingesting baits than younger more susceptible individuals.

Culling is usually the most simple and economical measure to control diseases spread in wildlife and its application is strongly supported as an emergency procedure for disease eradication. It has been historically applied to control different domestic and wildlife diseases with the aim of reducing host population or removing infected individuals, such as for bovine tuberculosis in badgers [13] and foot-and-mouth disease in cattle in the UK [14], avian flu in waterfowl birds and poultry in Asia [9] and classical swine fever in wild boars and domestic pigs in Europe [15]. Albeit, sport hunting *per se* is certainly not aimed at preventing disease spread, it usually exerts effects similar to those of culling in terms of population density reduction. The same is true also for illegal hunting (poaching).

Despite its simplicity and alleged cost effectiveness, there is some evidence that under a variety of circumstances culling may not generate the benefits anticipated [16]–[18]. Selective hunting may interfere with establishment of herd immunity inducing faster turnover of the population and decreasing host life expectancy. Furthermore, it may induce long distance host movement, increasing contacts between different groups of animals [19], and it may affect the evolution of some host species traits [20]. Finally, culling often affects age/stage structure by preferentially removing older and less susceptible individuals. This is important, as the basic theory for Susceptible-Infected-Recovery (SIR-like) compartmental models of a self-regulating host suggests that disease prevalence in a homogenous population should monotonically decrease with increasing culling rate [as in 21], [22 models]. However, as we will see, the existence of age/stage structure in transmission rate may significantly alter this conclusion

In the present work we show (using very general assumptions about life history traits of host species) that the presence of age-dependent heterogeneity in the transmission rate may produce the counter-intuitive result that disease prevalence increases over a range of intermediate levels of culling. We recast a simple SIR model for wildlife disease with two age/stage classes, namely, pre-reproductive juveniles and adults and sub-adults (see Material and Methods). Numerical characterization of the problem was examined for the specific case of classical swine fever in wild boar (see Protocol S1 in Supporting Information). We chose CSF as reference disease because it has caused serious economic losses in Europe from spillover infection to pig farms over the last twenty years [23] and it is still endemic in Asia and South America and in some regions of Eastern Europe and Italy (Sardinia). Also, Choisy and Rohani [24] have recently illustrated similar points using CSF but with a different model structure. Comparison across model structures for the same disease will lead to an increased robustness in any general characterization of optimal strategies for control.

While the ideas being investigated in the present work are based on a detailed understanding of wild boar biology, the epidemiological model itself has been simplified to address the following key question: how do age-dependent heterogeneities in transmission interact with culling rate in the control of disease prevalence?

To answer this question, we extend the classical SIR model of infection to allow for an age structured wildlife population. In this simple model, host population is divided into two age classes, juveniles and adults. We assumed that juveniles are highly susceptible to infection with associated high mortality and negligible recovery rates. On the other hand, infected adults and sub-adults exhibit negligible mortality and can recover with life-long immunity (see Material and Methods for details).

## Results

We have computed disease prevalence in the population at equilibrium as a function of culling rate or hunting mortality for different values of age-dependent heterogeneity in transmission, called *Δβ* = *β_a_*−*β_j_* (where, *β_a_* is the transmission rate between adults and *β_j_* is the transmission rate between juveniles). We held constant the basic reproduction number *R*_0_ in model (1) to keep the same level of infection in the population for each value of *Δβ* when culling is absent (see details in Protocol S2 in Supporting Information).

**Figure 1:**
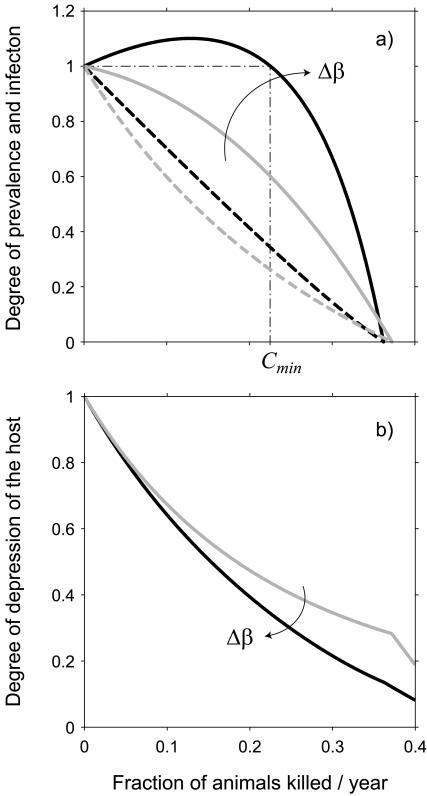
Effects of age/stage transmission on culling. a) Solid lines represent disease prevalence as function of the fraction of animals killed through culling scaled with respect to prevalence at equilibrium in the absence of culling; dotted lines refer to the number of infected individuals as functions of of the fraction of animals killed through culling scaled with respect to prevalence at equilibrium in the absence of culling; gray lines *Δβ* = 0 (*β_a_* = *β_j_* = 0.2856); black lines *Δβ* = *β_a_*−*β_j_* = 0.31 (*β_a_* = 0.32; *β_j_* = 0.01). In both cases *R*_0_ = 9.-b) Degree of depression of population abundance as a function of culling rate *c* under the same condition than above. Other parameter values have been set as follows: *ν* = 1.25 years^−1^, *μ_j_* = 0.9 years^−1^, *μ_a_* = 0.4 years^−1^, *γ* = 0.0067 (#individual^−1^ * 220 km^2^ * years^−1^), *α* = 25 years^−1^, *ρ* = 2 years^−1^, *δ* = 17.4 years^−1^.

When the within class transmission rate for adults is larger than that within juveniles, the disease prevalence can actually increase with culling or hunting rate instead of decreasing as expected under the assumption of homogenous mixing (Fig. 1a); disease prevalence eventually peaks for intermediate values of culling and then decreases only for high level of animal removal. Moreover, the absolute number of infected individuals is larger in the case of heterogeneous mixing relative to the number under conditions of homogenous mixing at all levels of culling. The explanation for this effect is that at low and intermediate levels of culling, by removing older resistant individuals, population age structure is skewed in favor of highly susceptible juvenile hosts thus making culling ineffective. The minimum culling rate *C_min_* required to bring disease prevalence below the value attained in the absence of culling can be fairly high, as depicted in Fig. 1a. As a consequence, as long as *c*<*C_min_*, culling actually performs worse, in terms of disease control, than the do-nothing alternative (*c* = 0). The dynamics of infection also reveal that the population density at equilibrium decreases with culling more quickly in the presence of in transmission heterogeneity (see Fig. 1b) even though, for intermediate values of culling, the actual number of infected hosts is larger in the case of heterogeneous transmission.

**Figure 2:**
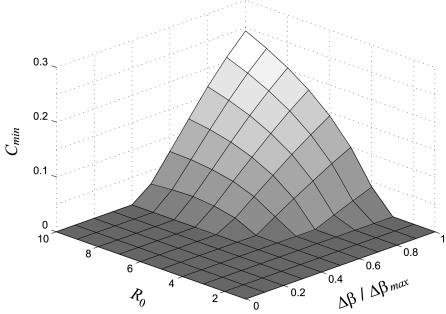
Value of the minimum culling rate *C_min_* required for disease prevalence to drop the value attained in absence of culling as a function of the basic reproduction number (*R*_0_) and the age-dependent heterogeneity in transmission (*Δβ* = *β_a_*−*β_j_*) renormalized by the maximum heterogeneities in transmission (*Δβ_max_*) allowed at each level of *R*_0_ (see Protocol S2).

We also performed a sensitivity analysis in the transmission parameters by estimating the values of *C_min_* for a broad range of *R*_0_ and *Δβ* values. Fig. 2 shows that negative effects of culling (corresponding to *C_min_*>0) can be important when both basic reproduction number and transmission heterogeneity are sufficiently large, otherwise the model predicts a monotonic decrease in prevalence with the culling rate (*C_min_* = 0).

## Discussion

The pattern revealed in Fig. 1a suggests that if the culling rate for disease eradication is computed by assuming homogenous mixing while transmission rate is actually age dependent, then classical culling strategies may prove to be ineffective: in fact, even though the host population is even more depressed then expected under homogeneous mixing, not only will the disease not be eradicated from the population but prevalence can be even higher than in the absence of culling. As a consequence, unless it is possible to guarantee a sufficiently high removal of adult individuals, the do-nothing alternative is more effective and less costly than an intermediate culling strategy. For example, in the case of classical swine fever, to move from *c* = 0 to *C_min_* requires removing at least 22.5% of individuals in the population. Similarly, intermediate levels of hunting pressure, especially when not aimed at disease control, might actually increase disease prevalence as well as the number of infected individuals. We show that similar counter-intuitive results are possible only in the presence of heterogeneity in transmission and sufficiently large *R*_0_ values (see Fig. 2).

On the other hand, the maintenance of a high level of culling that guarantees disease eradication is not always feasible in practice. In fact, if the host population is very small, culling might generate conservation concern, as the removal of a large fraction of individuals might drive the host to the brink of extinction, as argued by Dobson&Meagher [25] for the eradication of Brucellosis in Yellowstone National Park bison. On the contrary, if the host population is very large, it might be impossible to cull a large enough fraction of individuals. This may correspond to the case, for instance, for huge colonies of bats in central Africa suspected of being the reservoir of Ebola and Marburg viruses [26] or populations of small rodents in North America, that comprise the main reservoirs for Hanta viruses or the vectors of Lyme disease [27].

The results presented in Fig. 1 contain an important insight concerning the potential for disease to spill-over into domestic animals. If transmission between wildlife and domestic animals is density-dependent, the risk of spill-over decreases for increasing culling rates even though it is higher than in the case of homogenous mixing (dotted lines in Fig. 1a); while if transmission between wildlife and domestic animals is frequency dependent, the risk of spillover can substantially increase for intermediate values of culling rate with respect to the case of homogenous mixing (solid lines in Fig. 1a). Therefore, it is crucial to articulate and understand the exact mechanisms by which infectious wild hosts interact with susceptible domestic animals and humans susceptible in order to predict the effects of wildlife culling on the risks of spillover.

We thus conclude that age/stage structure of transmission rate can be crucial in our understanding of disease pattern and the implementation of control policies. This conclusion likely applies to other wildlife diseases in which age/size structure is an important component of population dynamics.

Recently, Choisy and Rohani [24] presented a model of wildlife disease that focused on the effects of strong density-dependence and seasonality on culling and equilibrium disease prevalence. Their model was different from ours in that it was not age-structured and culling was random over the population of hosts. Nonetheless, they showed a similar response to culling with intermediate levels of culling producing a counter-intuitive increase in disease prevalence. Given the explicit difference in model structures it is instructive to speculate on how two very different models can generate the same over all effect. As Choisy and Rohani indicate, the result of their model is driven by culling releasing the population from density-dependent reductions in the birth rate thereby producing a flush in new susceptible in the population. In our model we have a similar effect but driven by a completely different mechanism. The age-dependent culling coupled to the intrinsic heterogeneity in transmission similarly produces a flush in the relative abundance of the young susceptible class. We imagine that other mechanisms besides age/stage structure (as in our model) or strong density-dependence and seasonality (as in the Choisy and Rohani model), may interact with culling to produce similar patterns of prevalence.

Given the implications of transmission heterogeneities on dynamics and control, more detailed studies are thus necessary to assess these heterogeneities when structuring control strategies in current and ongoing wildlife epizootics [13], [16], [22]. What appears certain is that the negative effects of culling are robust to alternative model formulations and highlight the importance of better assessing transmission structure, seasonality, and population regulatory processes in the design of wildlife disease control strategies.

## Materials and Methods

The model is characterized by five classes: susceptible juveniles and adults, infected juveniles and adults and recovered (immune) adults.

The infection dynamics in this age-structured population are described by:
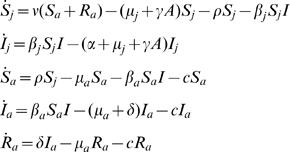
(1)where *S_j(a)_*, *I_j(a)_*, and *R_a_* refer to susceptible juveniles (adults), infected juveniles (adults), and recovered adults, respectively. The system parameters *ν*, *μ_j_*, *μ_a_*, and *ρ* represent the host birth rate, the juveniles mortality rate at low population density, the adults mortality rate, and the rate at which juveniles pass into adulthood, respectively. We assume that host population is self-regulating with density-dependent mortality in juveniles (*γ*) affected by total adults density (*A = S_a_+I_a_+R_a_*). The force of infection for susceptible juveniles (adults) individuals is *λ_j(a)_* =  *β_j(a)_I*, where *I = I_j_+I_a_* is the total infectious density in the population. Parameters *α* and *δ* represent the disease-induced mortality in juveniles, and the adult recovery rate, respectively. Finally, *c* is the control parameter and represents the culling effort over the adults population. Only adult and sub-adult individuals are here assumed to be culled either because of conservation measures or, in the case of hunting, because of the preference for large trophies. Moreover, culling is not usually allowed in spring when peak fertility occurs and when most juveniles are born. By the onset of the hunting season, at the beginning of autumn, juveniles have already moved into the sub-adult age class.

Contact rate among adults increases dramatically during mating season (when males roam considerable distances in search of reproductive females). Consequently, within adult transmission rate is assumed to be larger than within juvenile transmission. It is this variation in the within-class transmission rates that forms the basis of transmission heterogeneity. We assign the magnitude of this heterogeneity in age-specific transmission as a new variable, *Δβ* = *β_a_*−*β_j_* (≥0) assessing the marginal increase of adult transmission rate (*β_a_*) relative to that of juveniles (*β_j_*).

## Supporting Information

Protocol S1 Parameter setting for CSF. Details about the setting of epidemiological and demographic parameters in model (1) for classical swine fever in wild boar.
(0.04 MB DOC)

Protocol S2 Basic reproduction number computation. Details about calculation of the basic reproduction number for model (1).
(0.05 MB DOC)

## References

[1] Castillo-Chavez C (1989). Mathematical and Statistical Approaches to AIDS epidemiology..

[2] Hethcote HW, Van Ark JW (1992). Modeling HIV transmission and AIDS in the United States..

[3] Lloyd-Smith JO, Schreiber SJ, Kopp PE, Getz WM (2005). Superspreading and the effect of individual variation on disease emergence.. Nature.

[4] Galvani AP, May RM (2005). Dimensions of superspreading.. Nature.

[5] Heesterbeek JAP, Roberts MG, Grenfell BT, Dobson AP (1995). Mathematical models for microparasite of wildlife.. Ecology of Infectious Diseases in Natural Populations.

[6] Matthews L, Low JC, Gally DL, Pearce MC, Mellor DJ (2006). Heterogeneous shedding of *Escherichia coli* O157 in cattle and its implications for control.. Proceedings of the National Academy of Sciences of the United States of America.

[7] Grenfell BT, Dobson AP (1995). Ecology of Infectious Diseases in Natural Populations..

[8] Gilbert M, Mitchell A, Bourn D, Mawdsley J, Cliton-Hadley R (2005). Cattle movements and bovine tuberculosis in Great Britain.. Nature.

[9] Ellis TM, Bousfield RB, Bissett LA, Dyrting KC, Luk GSM (2004). Investigation of outbreaks of highly pathogenic H5N1 avian influenza in waterfowl and wild birds in Hong Kong in late 2002.. Avian Pathology.

[10] Real LA, Russell C, Waller L, Smith D, Childs J (2005). Spatial dynamics and molecular ecology of North American rabies.. Journal of Heredity.

[11] Sidwa TJ, Wilson PJ, Moore GM, Oertli EH, Hicks BN (2005). Evaluation of oral rabies vaccination programs for control of rabies epizootics in coyotes and gray foxes: 1995-2003.. JAVMA-Journal of the American Veterinary Medical Association.

[12] Kaden V, Hänel A, Renner C, Gosser K (2005). Oral immunisation of wild boar against classical swine fever in Baden-Württemberg: development of seroprevalences based on the hunting bags.. European Journal of Wildlife Research.

[13] Donnelly CA, Woodroffe R, Cox DR, Bourne J, Cheeseman CL (2006). Positive and negative effects of widespread badger culling on tuberculosis in cattle.. Nature.

[14] Haydon DT, Kao RR, Kitching RP (2004). The UK foot-and-mouth disease outbreak–the aftermath.. Nature Reviews Microbiology.

[15] Pluimers FH, de Leeuw PW, Smak JA, Elbers ARW, Stegeman JA (1999). Classical swine fever in The Netherlands 1997-1998: a description of organisation and measures to eradicate the disease.. Preventive Veterinary Medicine.

[16] Donnelly CA, Woodroffe R, Cox DR, Bourne J, Gettingby G (2003). Impact of localized badger culling on tuberculosis incidence in British cattle.. Nature.

[17] Shirley MDF, Rushton SP (2005). Where diseases and networks collide: lessons to be learnt from a study of the 2001 foot-and-mouth disease epidemic.. Epidemiology and Infection.

[18] Woodroffe R, Cleaveland S, Courtenay O, Laurenson MK, Artois M, Macdonald DW, Sillero-Zubiri C (2004). Biology and conservation of wild canids.. Biology and conservation of wild canids.

[19] Laddomada A (2000). Incidence and control of CSF in wild boar in Europe.. Veterinary Microbiology.

[20] Coltman DW, O'Donoghue P, Jorgenson JT, Hogg JT, Strobeck C (2003). Undesirable evolutionary consequences of trophy hunting.. Nature.

[21] Anderson RM, Jackson HC, May RM, Smith AM (1981). Population dynamics of rabies in Europe.. Nature.

[22] Coyne MJ, Smith G, McAllister FE (1989). Mathematical model for the population biology of rabies in raccoons in the mid-Atlantic states.. American Journal of Veterinary Research.

[23] Saatkamp HW, Dijkhuizen AA, Geers R, Huirne RBM, Noordhuizen JPTM (1997). Economic evaluation of national identification and recording systems for pigs in Belgium.. Preventive Veterinary Medicine.

[24] Choisy M, Rohani P (2006). Harvesting can increase severity of wildlife disease epidemics.. Proceedings of the Royal Society B-Biological Sciences.

[25] Dobson A, Meagher M (1996). The population dynamics of Brucellosis in the Yellowstone National Park.. Ecology.

[26] Leroy EM, Kumulungui B, Pourrut X, Rouquet P, Hassanin A (2005). Fruits bats as reservoirs of Ebola virus.. Nature.

[27] Oliver JH, Lin T, Gao L, Clark KL, Banks CW (2003). An enzootic transmission cycle of Lyme borreliosis spirochetes in the southeastern United States.. Proceedings of the National Academy of Sciences of the United States of America.

