# Supplementary material for: Transmission Heterogeneity and Control Strategies for Infectious Disease Emergence
Source: PLoS One. 2007 Aug 22;2(8):e747. doi: 10.1371/journal.pone.0000747 (PMC1945090; doi:10.1371/journal.pone.0000747)
Supplement: Protocol S1 — Parameter setting for CSF. Details about the setting of epidemiological and demographic parameters in model (1) for classical swine fever in wild boar. (0.04 MB DOC) [file pone.0000747.s001.doc]

# PROTOCOL S1:

Details about the setting of epidemiological and demographic parameters in model (1) for classical swine fever in wild boar:

Wild boar populations are characterized by a well-defined social structure with closely related adult females (1 years old or more) and sub-adult females (6-12 months old) grouping together with their piglets (0-6 months old). Young males and females become sub-adults after about six months. Sub-adult males leave the herd and roam around in search of new territory in the fall at the beginning of the hunting season. Adult males tend to be more sedentary but they wander over considerable distances during the mating season, thus greatly increasing their contact rate with respect to that of piglets. This difference in movement patterns between piglets and adult individuals may obviously affect disease transmission among different age classes similarly to what has been observed for humans (see for example [1]).

Vital parameters of the host population have been set in accordance with field studies on the ecology of wild boar: Fenati and Guberti estimated that potential number of daughters produced by an adult sow per year is about 2.5 individuals/year (Fenati, M. & Guberti, V., unpublished data). As the observed rate of increase of the population at low densities in absences of harvesting is about 2.5, we have set the birth rate ν at 1.25 year^-1^. Bieber & Ruf [2] estimated that piglets survival in the first year of life ranges between 25-52% with mean value around 40%. As a consequences the natural mortality rate at low density μ*_j_* is equal to 0.9 year^-1^ for individuals with age<6 months. The parameter μ*_a_* (adult mortality rate) can be easily calibrated as the inverse of the average life expectancy of an adult host, namely about 2-3 years. Guberti et al. [3] estimated the carrying capacity of a Sardinian wild boar population in 600 individuals on a surface of 220 km^2^ which yield to instraspecific competition coefficient γ equal to 0.0067 (220 km^2^ years^-1^).

The main means of viral transmission is by direct contact between infected and susceptible animals.

Once infected, wild boars become infectious after a short latent period of 2-6 days. Young individuals die between 10 and 20 days post-infection, while most adults recover after 3 weeks. Even though the issue is very controversial, wild boar have been considered the reservoir of the disease [4] and, consequently, eradication policies in recent years have been based essentially on vaccination and culling, that is, the removal of animals to push population density below the threshold for disease invasion Culling has been widely implemented throughout Europe in the 1990s and is still considered a central component of national control plans of the disease in the wildlife in many Member States of the European Union [5].

Disease induced mortality in piglets (α) has been estimated as the inverse of the average time spent by a juvenile in the infected class before dying, that is about 15 days. The recovery rate of infected adults wild boar (δ) has been estimated as the inverse of the average time spent by adult in the infectious class *I_a_* before recovering, namely about 3 weeks.

Howard & Donnelly [6] estimated the basic reproduction number (*R_0_*) of a wild boar CSF infection in Pakistan. Through the epidemic wave, *R_0_* ranges from values under unity to picks larger than 10. In our analyses we have set *R_0_*=9 as this figure well fits the range of observed basic reproduction numbers of CSF in wild boar estimated for Italian populations by Guberti (pers. comm.).

**Protocol S1 References:**

1. Anderson, R.M. and R.M. May, *Infectious Diseases of Humans: Dynamics and Control*. 1991, Oxford: Oxford Science Publications.

2. Bieber, C. and T. Ruf, *Population dynamics in wild boar sus scrofa: ecology, elasticity of growth rate and implications for the management of pulsed resource consumers.* Journal of Applied Ecology, 2005. **42**: p. 1203-1213.

3. Guberti, V., et al. *Estimate threshold abundance for the persistence of the classical swine fever in the wild boar population in Eastern Sardinia*. in *Measures to control classical swine fever in European wild boar*. 1998. Perugia, Italy.

4. Laddomada, A., *Incidence and control of CSF in wild boar in Europe.* Veterinary Microbiology, 2000. **73**: p. 121-130.

5. Laddomada, A., *Classical swine fever in wild boar*. 1999, Directorate-General XXIV, Scientific Committee on Animal Health and Animal Welfare, XXIV/B3/R09/1999: Brussels, Belgium.

6. Howard, S.C. and C.A. Donnelly, *Estimation of a time varying force of infection and basic reproduction number with application to an outbreak of classical swine fever.* Journal of Epidemiology and Biostatistics, 2000. **5**: p. 161-168.
