# Supplementary material for: Transmission Heterogeneity and Control Strategies for Infectious Disease Emergence
Source: PLoS One. 2007 Aug 22;2(8):e747. doi: 10.1371/journal.pone.0000747 (PMC1945090; doi:10.1371/journal.pone.0000747)
Supplement: Protocol S2 — Basic reproduction number computation. Details about calculation of the basic reproduction number for model (1). (0.05 MB DOC) [file pone.0000747.s002.doc]

# PROTOCOL S2:

*Details about calculation of the basic reproduction number for model (1):*

We calculated the basic reproduction number *R*_0_ for model (1) as a function of within-class disease transmission *β_j_* and *β_a_*.

The basic reproduction number for heterogeneous population, whose individuals are distinguishable by age or behavior, but can be grouped in homogeneous compartments, may be calculated as suggested by van den Driessche & Watmough [1]. They define the basic reproduction number as the spectral radius of the ‘next generation’ matrix (*FV^-1^*).

*R_0_ = ρ*(*FV^-1^*)

where *F* and *V* are defined as Jacobian matrix of new infections appearance matrix F and the Jacobian matrix of other rates of transfer matrix V calculated for infected compartments in the disease-free equilibrium (DFE) *x_0_* = [*K_j_*(*c*), 0, *K_a_*(*c*), 0, 0]^T^. Where *K_j_*(*c*) and *K_a_*(*c*) are the carrying capacities of juveniles and adults.

F $=\left[\begin{matrix} \begin{matrix} 0 \\ \beta_{j}S_{j}I \\ 0 \\ \beta_{a}S_{a}I \end{matrix} \\ 0 \end{matrix}\right]$ and V $=\left[\begin{matrix} \begin{matrix} -\nu(S_{a}+R_{a})+(\mu_{j}+\gamma A)S_{j}+\rho S_{j}+\beta_{j}S_{j}I \\ (\alpha+\mu_{j}+\gamma A)I_{j} \\ \mu_{a}S_{a}+\beta_{a}S_{a}I+cS_{a}-\rho S_{j} \\ (\mu_{a}+\delta)I_{a}+cI_{a} \end{matrix} \\ \mu_{a}R_{a}+cR_{a}-\delta I_{a} \end{matrix}\right]$

The infected compartments are *I*_j_ and *I*_a_, hence:

$F=\left[\begin{matrix} \beta_{j}K_{j}(c) & \beta_{j}K_{j}(c) \\ \beta_{a}K_{a}(c) & \beta_{a}K_{a}(c) \end{matrix}\right]$ and $V=\left[\begin{matrix} \alpha+\mu+\gamma K_{a}(c) & 0 \\ 0 & \mu_{a}+\delta+c \end{matrix}\right]$

with *K_j_* and *K_a_* monotonically decreasing functions of the culling rate:

$$K_{j}=\left[\nu-(\mu_{a}+c)(\mu_{j}/\rho)\right]/\gamma$$

$$K_{a}=\left[\rho\nu-(\mu_{a}+c)(\mu_{j}+\rho)\right]/\left[\gamma(\mu_{a}+c)\right]$$

The next generation matrix is:

$$FV^{-1}=\frac{1}{(\alpha+\mu_{j}+\gamma K_{a})(\mu_{a}+\delta+c)}\left[\begin{matrix} \beta_{j}K_{j}(\mu_{a}+\delta+c) & \beta_{j}K_{j}(\alpha+\mu_{j}+\gamma K_{a}) \\ \beta_{a}K_{a}(\mu_{a}+\delta+c) & \beta_{a}K_{a}(\alpha+\mu_{j}+\gamma K_{a}) \end{matrix}\right]$$

and its spectral radius is:

$$R_{0}=\rho(FV^{-1})=\frac{\beta_{j}K_{j}(c)}{(\alpha+\mu_{j}+\gamma K_{a}(c))}+\frac{\beta_{a}K_{a}(c)}{(\mu_{a}+\delta+c)}$$

Then, the basic reproduction number in host population is sum of the contribution of juveniles and adults to infection.

As a consequence, for a fixed value of *R*_0_, the age-dependent heterogeneity in transmission (*Δβ *= *β_a_* − *β_j_*) can range between the finite values of *Δβ_min_* (when *β_a_* = 0) and *Δβ_max_* (when *β_j_* = 0); at which correspond values of *β_j_* = *R*_0_ (*α *+ *μ_j_* + *γ K_a_*(*c*)) / *K_j_*(c) and *β_a_* = *R*_0_ (*μ_a_* + *δ *+ *c*) / *K_a_*(*c*), respectively.

**Protocol S2 References:**

1. van den Driessche P, Watmough J (2002) Reproduction numbers and sub-threshold endemic equilibria for compartmental models of disease transmission. Mathematical Biosciences 180: 29-48.
